# Targeting PRAME for acute myeloid leukemia therapy

**DOI:** 10.3389/fimmu.2024.1378277

**Published:** 2024-03-26

**Authors:** Jinjun Yang, Mengran Chen, Jing Ye, Hongbing Ma

**Affiliations:** ^1^ Department of Hematology and Institute of Hematology, West China Hospital, Sichuan University, Chengdu, China; ^2^ Department of Dermatology and State Key Laboratory of Biotherapy, West China Hospital, Sichuan University, Chengdu, China

**Keywords:** PRAME, acute myeloid leukemia, leukemia-associated antigen, minimal residual disease, immunotherapy, adoptive T-cell therapy

## Abstract

Despite significant progress in targeted therapy for acute myeloid leukemia (AML), clinical outcomes are disappointing for elderly patients, patients with less fit disease characteristics, and patients with adverse disease risk characteristics. Over the past 10 years, adaptive T-cell immunotherapy has been recognized as a strategy for treating various malignant tumors. However, it has faced significant challenges in AML, primarily because myeloid blasts do not contain unique surface antigens. The preferentially expressed antigen in melanoma (PRAME), a cancer-testis antigen, is abnormally expressed in AML and does not exist in normal hematopoietic cells. Accumulating evidence has demonstrated that PRAME is a useful target for treating AML. This paper reviews the structure and function of PRAME, its effects on normal cells and AML blasts, its implications in prognosis and follow-up, and its use in antigen-specific immunotherapy for AML.

## 1 Introduction

In adults, acute myeloid leukemia (AML) is the most common heterogeneous acute leukemia, and it is typically associated with a poor prognosis (1). For more than 40 years, the induction of intensified chemotherapy (i.e., “7+3”) based on cytarabine and anthracycline drugs has been the standard treatment for AML. Since 2017, there has been a rapid expansion in the utilization of anti-AML medications following years of limited progress in the approval of new drugs (2). For some patients with specific genetic mutations, such as those harboring mutations in FLT3, IDH1, and IDH2, targeted drugs can be chosen for treatment. However, there are still many patients who do not have relevant molecular mutation targets. One of the most revolutionary treatments is the combination of azacytidine and venetoclax, which has almost completely replaced traditional cytotoxic chemotherapy in many cases. However, there is a poor prognosis for patients who progress on azacytidine/venetoclax, particularly if they have mutations in TP53. Therefore, it is crucial to develop additional treatment options in clinical practice.

In the past decade, there have been breakthroughs in adaptive T-cell immunotherapy for hematologic malignancies and solid tumors (3). However, immunotherapy for AML is limited by congenital heterogeneity and the lack of specific surface antigens on myeloid leukemia cells (4). Due to the high cross-expression of leukemia cells and hematopoietic stem/progenitor cells (HSPCs), the use of nonspecific lineage markers as therapeutic targets (e.g., CD33) may result in severe bone marrow suppression. The field of AML immunotherapy is increasingly focusing on specific leukemia-associated antigens (LAAs), including preferentially expressed antigen in melanoma (PRAME), CLL-1, TIM-3, and WT1 (5). Researchers have focused their attention on PRAME because it is expressed at high levels in AML but is absent from normal HSPCs.

The PRAME gene encodes the restricted antigenic peptide for the human leukocyte antigen HLA-A24. PRAME, also known as MAPE (melanoma antigen preferentially expressed in tumors), CT130 (cancer testis antigen 130), or OIP4 (Opa-interacting protein 4), was first identified in human melanoma cells by Ikeda et al. in 1997 (6). In addition to being expressed in melanoma patients, PRAME has also been found to be overexpressed in AML patients but not in normal hematopoietic tissues. More importantly, PRAME is also expressed on leukemic stem/progenitor cells (LSCs/LPCs), which are self-renewing cells that can produce many daughter blasts, a major cause of leukemia relapse. In addition, the PRAME antigen can be recognized by autologous cytotoxic T lymphocytes (CTLs). These characteristics make PRAME a promising target for vaccination studies and adoptive antileukemia immunotherapy for AML. The purpose of this paper is to review the current advancements in PRAME, its structure and function, its effects, its expression, and its clinical implications in AML.

## 2 The structure and function of PRAME

**Figure 1:**
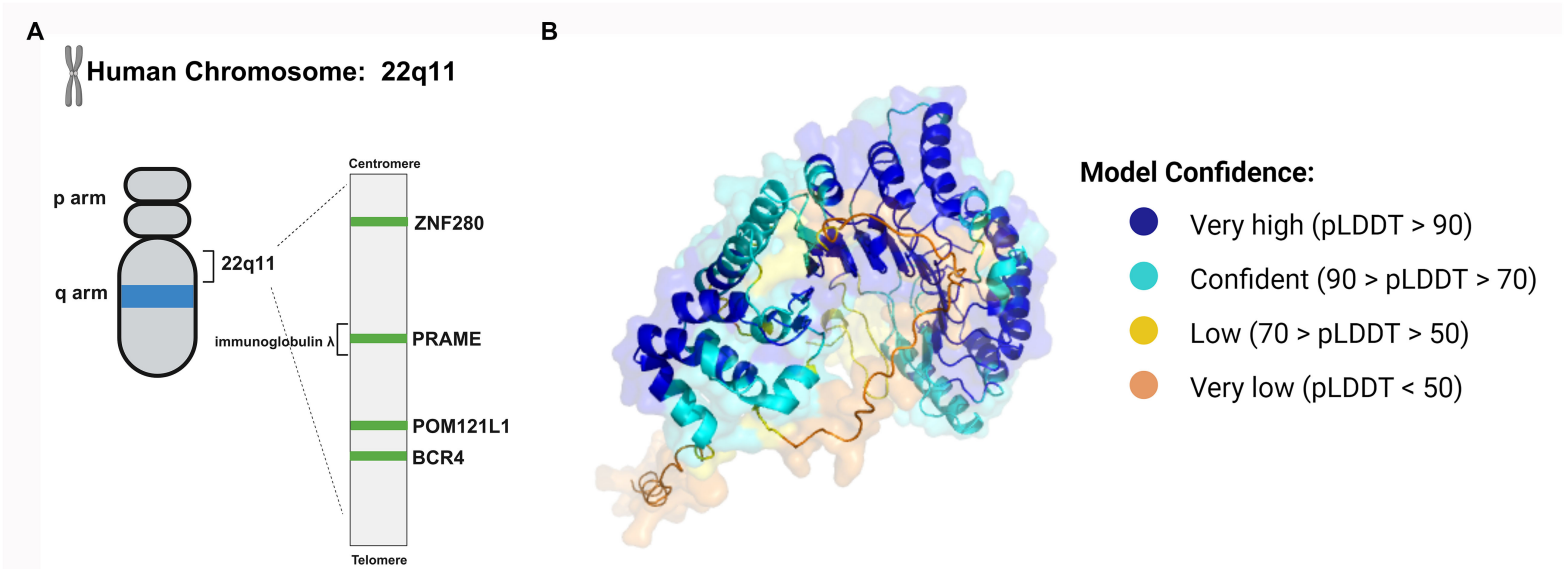
The structure of the PRAME gene and protein. **(A)** The human PRAME gene is located on chromosome 22q11 and between ZNF280 and POM121. **(B)** The PRAME protein contains leucine-rich repeat (LRR) sequences. The LRRs form a β sheet and then an α-helix, and the repeating units can induce a curved solenoid fold. The three-dimensional conformation of PRAME was computationally modeled by AlphaFold (https://alphafold.ebi.ac.uk/entry/P78395), which assigns a per-residue confidence score known as pLDDT, ranging from 0 to 100. Residues are color-coded based on their respective pLDDT values to visually represent the varying levels of prediction confidence.

As a member of the PRAME multigene family, PRAME can be found in humans and other mammals. The PRAME gene appears to have replicated multiple times during human evolution, and the human genome contains at least 22 PRAME-like genes and 10 pseudogenes (7). The human PRAME gene consists of approximately 12 kilobases and is located on chromosome 22q11.22 (**Figure 1A**). It is located in the human immunoglobulin λ gene locus and is crucial for the generation of λ light chains during B-cell development (8). There are several other nonimmunoglobulin genes at this locus, including ZNF280 and POM121L1. POM121L1 is adjacent to BCR4, which has been identified as a breakpoint cluster associated with some rearrangements on chromosome 22 (**Figure 1A**).

The PRAME mRNA contains 6 exons and encodes a membrane-binding protein containing 509 amino acids. The PRAME protein contains leucine-rich repeat sequences (LRRs), which consist of approximately 20-30 amino acids, 21.8% of which are leucine or isoleucine (9). LRRs form a β sheet and then an α-helix, and the repeating units can induce a curved solenoid fold (10). Nevertheless, not all LRRs fold in this way. For instance, PRAME may fold similarly to the LRR domains of Toll-like receptors (TLRs) 3 and 4 and internalizing proteins (**Figure 1B**) (7, 11, 12). Its tertiary structure enables PRAME to interact with proteins, nucleic acids, and other ligands, thus playing an important role in cellular immunity, adhesion, and signal transduction.

As a cancer-testis antigen, PRAME is expressed mainly on the testes, ovaries, and endometrium in normal tissues (13, 14). The PRAME gene family is actively transcribed in the germline throughout life. It plays a pivotal role in germline development and gametogenesis, including maintaining embryonic stem cell pluripotency and participating in the proliferation and differentiation of germ cells as well as in the formation of the acrosome and sperm tail during spermiogenesis (15). PRAME is also overexpressed in some cancers, such as breast cancer, cervical cancer, lung cancer, ovarian cancer, melanoma, sarcoma and hematological malignancies, and its function in these cancers depends on the tumor type and downstream targets that mediate cell differentiation, proliferation, apoptosis, growth arrest and chemotherapy sensitivity (16–25).

**Figure 2:**
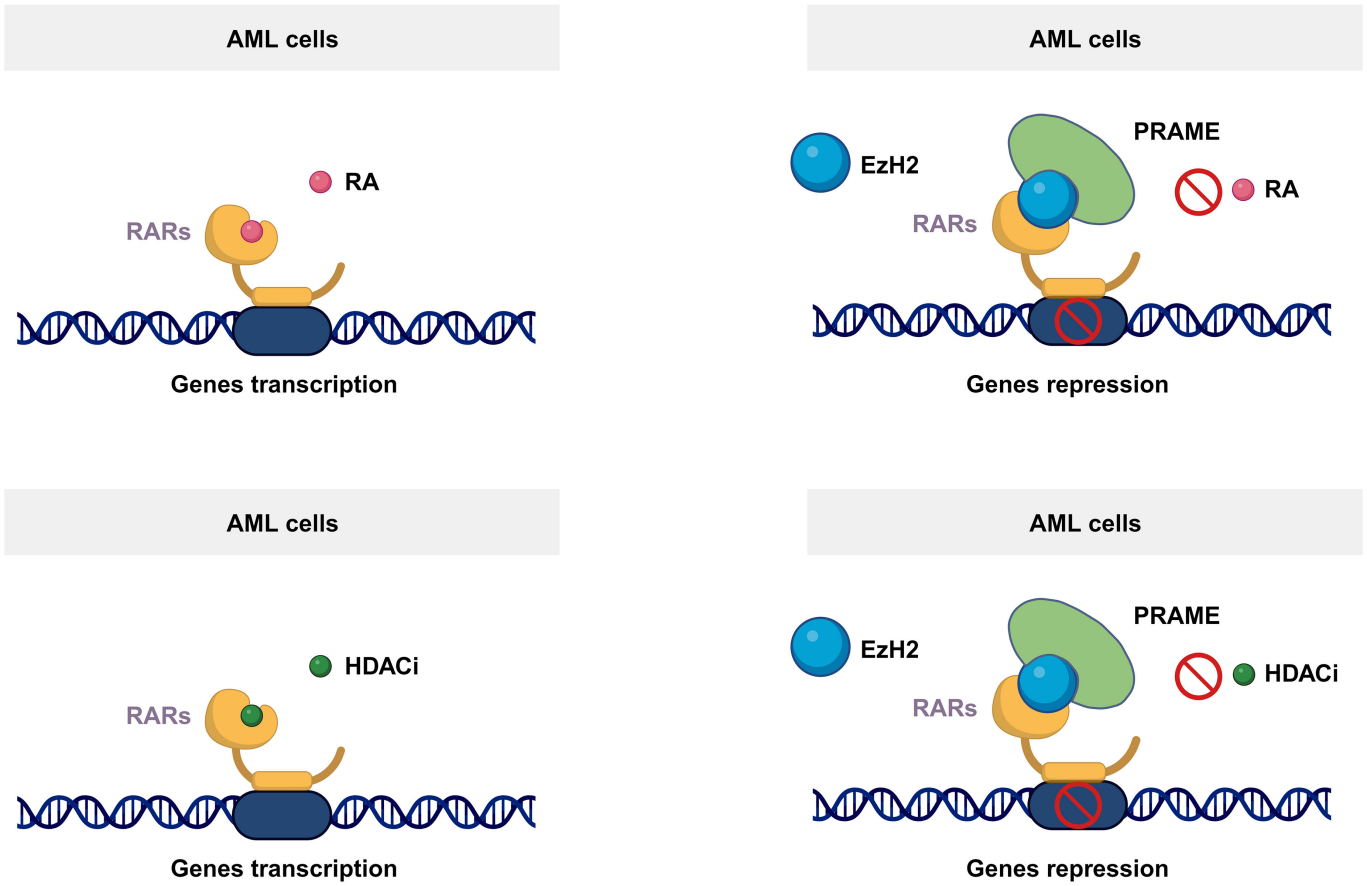
Mechanism of the PRAME function. As a ligand-dependent corepressor of RAR- and RAR-related signaling, PRAME binds to RARs and prevents receptor activation and transcription of target genes by recruiting EZH2. Additionally, PRAME inhibits RAR signaling mediated by histone deacetylase inhibitors, thereby affecting downstream gene transcription.

PRAME can act as a transcriptional repressor and inhibit retinoic acid receptor (RAR)-mediated growth arrest and differentiation (26). It is well known that RARs play essential roles in regulating the proliferation and differentiation of hematopoietic cells (27). As a ligand-dependent corepressor of RAR and RAR-related signaling, PRAME binds to RARs and prevents receptor activation and transcription of target genes (**Figure 2**) (26, 28). Consequently, it has been suggested that PRAME may contribute to AML disease progression by repressing RAR function (26). According to Wadelin et al., PRAME binds very weakly to RARs and other nuclear receptors. Therefore, indirect interactions with other proteins may facilitate functional interactions between PRAME and RARs (29). EZH2 is reportedly recruited by PRAME to suppress RAR signaling (**Figure 2**) (26). However, another study showed that PRAME expression does not contribute to the downregulation of RAR signaling in primary AML cells (30). This could be partially explained by the findings that PRAME inhibits myeloid differentiation in a retinoic acid-dependent or retinoic acid-independent manner (31). Therefore, the effect of PRAME on RAR signaling in AML cell lines and patients’ needs further validation. Additionally, PRAME inhibits RAR signaling mediated by histone deacetylase (HDAC) inhibitors, thereby affecting downstream gene transcription (**Figure 2**) (32).

**Figure 3:**
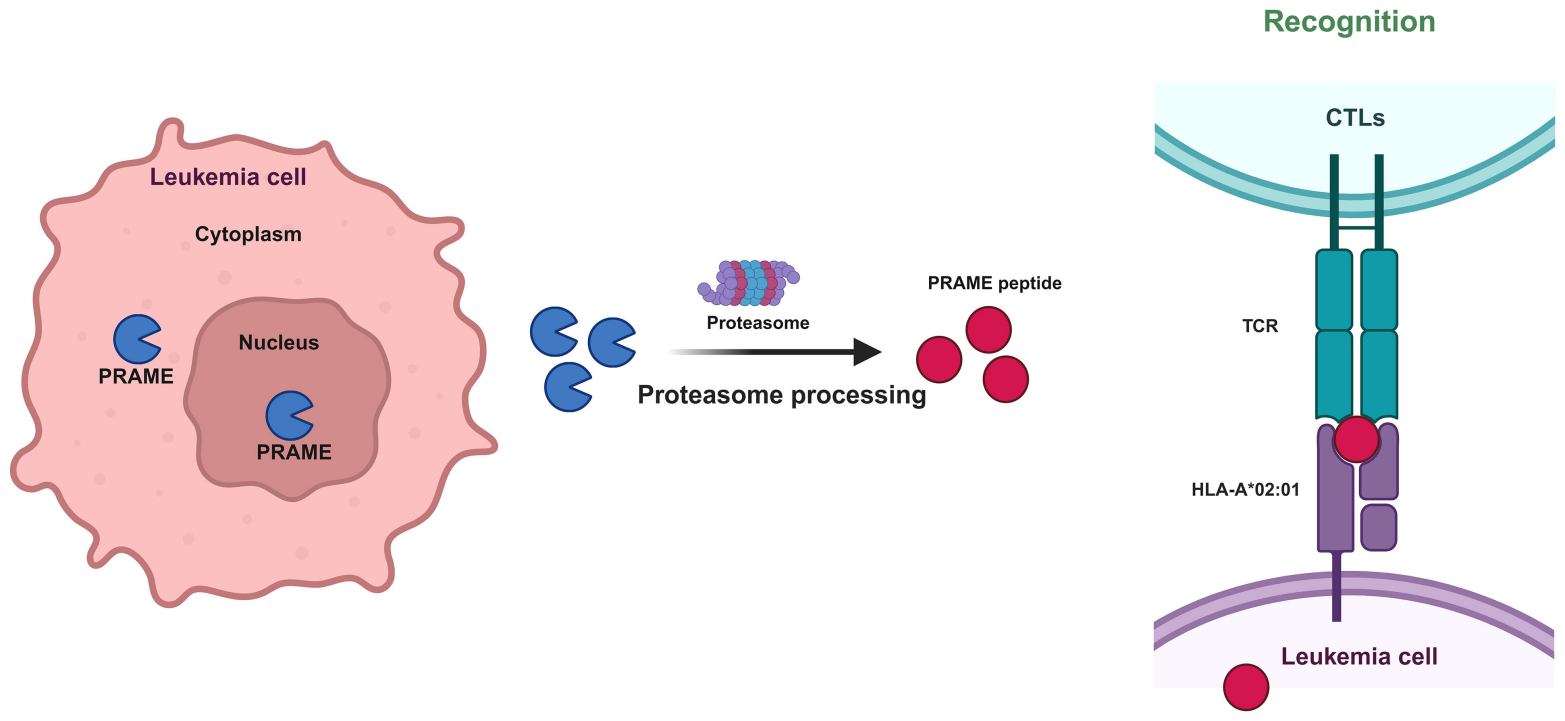
Mechanism by which CTLs recognize PRAME-mediated leukemia cells. Protease processing of the PRAME peptide results in recognition of HLA-A02:01 by the T-cell receptor (TCR) of CTLs.

As an intracellular protein, PRAME is found both within the nucleus and the perinuclear region (16). In accordance with its ability to localize to the nucleus, PRAME contains several nuclear localization signal sequences, such as 157-KKRKV-161 and 198-KVKRKKNV-205. When the PRAME peptide is protease-processed, HLA-A02:01 is recognized by the T-cell receptor (TCR) of CTLs after being processed by proteasomal enzymes (**Figure 3**). Based on this phenomenon, it is rational to utilize HLA-A*02–restricted PRAME-peptide to generate PRAME CTLs from healthy donors or patients, which could release IFNγ and lyse PRAME peptide–expressing cells in an MHC-restricted manner and pave the way for AML immunotherapy (33).

## 3 Expression of PRAME in normal hematopoietic and AML cells

PRAME is not present or is expressed at extremely low levels in the majority of normal tissues, such as the testis, ovary, adrenal, endometrium, placenta, bone marrow, CD34^+^ hematopoietic progenitors, unsorted peripheral blood cells, and sorted B and T lymphocytes (6, 31, 34–38). However, it is highly expressed in numerous types of human malignancies, including most primary and metastatic melanomas (6), ovarian cancer (16), breast carcinoma (39), lung carcinomas (40), neuroblastoma (41), cervical cancer (25), and head and neck cancers (42). Furthermore, PRAME is significantly increased in diverse hematologic malignancies, including acute and chronic leukemias, non-Hodgkin’s lymphomas, and multiple myeloma, distinguishing it from the majority of other tumor-associated antigens (TAAs) (34–36, 43–46).

Kirkey et al. examined the gene expression patterns of more than 2000 individuals, including children and young adults, diagnosed with AML in comparison to those with normal hematopoiesis. PRAME was found to be one of the genes with the highest level of expression in AML patients, but it was not detected in normal peripheral blood (PB) CD34^+^ and bone marrow (BM) samples (47). Similarly, Mumme et al. employed single-cell RNA sequencing to examine pediatric AML BM samples during diagnosis, induction completion, and relapse. Through this analysis, they discovered PRAME as a 7-gene signature (PRAME, CLEC11A, AZU1, NREP, ARMH1, C1QBP, and TRH) associated with AML blasts (48). PRAME is highly expressed in 30–64% of individuals diagnosed with AML (35–37, 49–54), and its expression level is linked to particular subcategories of AML. Multiple research studies have indicated that individuals diagnosed with AML and possessing t(8;21), del(7q)/-7, or t(15;17) chromosomal abnormalities tend to have elevated levels of PRAME (35, 36, 49, 50, 54–57). Conversely, patients with complex karyotypes or inv (16) display comparatively lower average PRAME levels (57). However, whether the fusion proteins AML1/ETO and PML/RARα directly or indirectly promote PRAME expression needs to be investigated. According to Greiner et al., PRAME expression was decreased in AML patients with a history of malignancy (*P* = 0.02) (57). Furthermore, PRAME expression was additionally recognized as a distinct discriminatory indicator that differentiates between transient and acute megakaryoblast leukemia in individuals with Down’s syndrome (58). In addition, compared to the chronic phase, there was a correlation between disease progression and heightened PRAME expression in patients experiencing blast crisis (31, 34, 59). Finally, the PRAME expression level was increased in relapsed patients compared to that in newly diagnosed patients (34).

The expression of PRAME is epigenetically regulated by DNA methylation (60, 61). In normal tissues, the PRAME gene is hypermethylated, whereas in the majority of malignant cells, this gene is hypomethylated. Administering the demethylating agent decitabine leads to a dose-dependent increase in PRAME expression in PRAME-deficient U937 and THP1 cell lines exhibiting PRAME hypermethylation, indicating that treatment with a demethylating agent induces an increase in PRAME expression in specific cancerous cells (60, 62).

## 4 Effects of PRAME on leukemia cells in AML

PRAME is an oncogene in many solid tumors and hematological tumors. However, the role of PRAME and its mechanism of action in AML cells remain controversial.

Several studies have suggested that PRAME is an oncogene in AML. Bullinger et al. showed that PRAME could inhibit differentiation mediated by RARs (28). Goellner et al. reported that apoptosis genes were expressed at lower levels in childhood patients with *no* AML who had increased PRAME expression (63). A study by Tanaka et al. showed that PRAME increased the proliferation of leukemia cells, promoted cell cycle progression, and inhibited apoptosis in leukemia cells (64). Additionally, PRAME inhibition altered the expression of genes involved in erythroid differentiation (64). Moreover, Oehler et al. showed that PRAME inhibited myeloid differentiation in HSPCs and LPCs (31). Finally, Tajeddine et al. showed that PRAME suppression could cause significantly decreased tumorigenicity in a xenograft mouse model, suggesting that PRAME could be a target for leukemia therapy (16).

However, other studies have shown that PRAME is a tumor suppressor gene in AML. PRAME expression might induce caspase-independent cell death, inhibit cell proliferation, induce apoptosis, and cause cell cycle arrest in leukemia cells (16, 65, 66). In addition, Xu et al. reported that repressing PRAME expression via short interfering RNA increased the tumorigenicity of K562 leukemic cells in nude mice (65).

These conflicting results concerning the function of PRAME in AML cells were similar to the data mentioned above for different cancer types. One of the possible reasons is that the function of PRAME is cell lineage dependent. Oehler et al. reported that PRAME could facilitate proliferation and inhibit ATRA-induced granulocytic differentiation in the HL60 and NB4 cell lines but could not restrain differentiation in K562 cells (31). Steinbach et al. showed that PRAME expression did not downregulate retinoid acid signaling in primary AML (30). The underlying mechanism related to cell type needs to be investigated in the future. In addition, different experimental conditions may also lead to contrary results even when the same cell line is used. Tanaka et al. and Tajeddine et al. utilized small interfering RNAs (siRNAs) to knock down PRAME in the K562 cell line, and tumor inhibition and growth were observed *in vitro* and *in vivo*, respectively (16, 64) Consequently, more studies are needed to further elucidate the opposing conclusions.

## 5 Prognosis implications

Several studies have indicated that elevated PRAME levels are linked to unfavorable results, resistance to medication, and disease progression in individuals with solid tumors (39, 41), chronic leukemia, Hodgkin’s lymphoma, multiple myeloma, and diffuse large B-cell lymphoma (17–19, 67). Nevertheless, in cases of pediatric AML, acute promyelocytic leukemia, and the majority of adult AML cases, enhanced PRAME expression is linked to improved prognosis. In a cohort of 28 pediatric AML patients, PRAME-positive patients (36%) exhibited significantly greater leukemia-free survival (LFS) than did PRAME-negative patients (53). In addition, Santamaría et al. established a molecular stratification model for prognosis in 121 patients with cytogenetically normal AML, and high PRAME, low ERG and low EVI1 were assigned as favorable parameters that were associated with longer relapse-free survival (RFS) and overall survival (OS). The 2-year OS and RFS in patients with high PRAME were 63% and 79%, respectively, while in patients with low PRAME, they were 51% and 48%, respectively. Patients who were refractory to induction chemotherapy had lower PRAME levels than patients who achieved CR after induction chemotherapy. There was an association between the overexpression of PRAME and a better response to induction chemotherapy. Interestingly, this stratification model identified PRAME patients with longer OS and RFS, even in the FLT3/NPM1 intermediate-risk/high-risk subgroup (68). Similar results were also observed in other studies (36, 55).

There are some opinions on why PRAME has a good prognosis. First, the good prognosis of PRAME may be related to the higher incidence rates of t(8;21) and t(15;17) in PRAME-positive AML patients (35, 52, 55). However, it has been argued that PRAME is an independent good prognostic factor among generally poor prognosis karyotypes, as indicated by del(7q)/-7 (57). Second, considering that autologous CTLs recognize PRAME, it can be inferred that the favorable prognosis and reduced load of leukemia blasts are a direct result of the immune reaction of PRAME. Third, the positive predictive value of PRAME in AML may be attributed to the suppression of S100A4 and the persistent stimulation of P53 activation (66). Ultimately, the expression of PRAME significantly affects the clinical outcomes of AML patients who undergo all-trans retinoic acid (ATRA) therapy. A significant clinical benefit was observed in younger AML patients with high PRAME expression who received ATRA treatment in comparison to all other patients (28, 69).

## 6 Minimal residual disease monitoring

Currently, the molecular minimal residual disease (MRD) targets applied to AML are restricted to oncogenic fusion transcripts, such as PML/RARα, RUNX1/RUNX1T1, CBFβ/MYH11, KMT2A/MLLT3 and NPM1 mutations (70–74). However, more sensitive modalities are lacking for up to 70% of AMLs, limiting the monitoring frequency (75). Studies have indicated that in the majority of AML patients, the expression of WT1 is notably high, providing an opportunity for MRD monitoring (76). However, WT1 is expressed by normal hematopoietic progenitors (77), and it has not been found to be reliable by some investigators (78), which limits its application. To enhance sensitivity and specificity, it will be beneficial to explore alternative genes that can be monitored alongside WT1. Because the PRAME gene is transcribed in leukemia cells and LSCs (79) but not in normal BM or PB mononuclear cells, quantitative real-time PCR (qPCR) using PRAME-specific oligonucleotides may be a valuable tool for detecting leukemia cells.

The PRAME could be used to determine the response to induction chemotherapy, evaluate the remission rate, detect MRD, and predict relapse. The high expression of PRAME in diagnosis and recurrence provides a foundation for monitoring MRD and predicting recurrence in PRAME-positive AML patients. Previously, studies confirmed that PRAME expression was suitable for monitoring MRD status by simultaneously assessing the quantitative expression of AML1/ETO or PML/RARα and PRAME (52, 55). The sensitivity of PRAME detection can reach 1 in 10^5^ patients (36). Multiple studies have demonstrated that PRAME levels decrease during remission and increase during relapse (49, 51, 54, 80). The results indicate that PRAME serves as a reliable indicator for identifying MRD status and detecting relapse prior to both morphologic and molecular relapse, particularly in individuals lacking identifiable genetic markers (49, 80, 81). In addition, some studies have shown that PRAME, along with other LAAs, can serve as a tool for monitoring MRD and predicting recurrence (38, 82). In addition to providing AML patients with either a positive or a more sensitive molecular marker for MRD monitoring, simultaneous detection of PRAME and other LAAs could also prevent false negatives (54). Steinbach et al. determined the prognostic relevance of monitoring MRD status via seven genes (including *PRAME*) in a prospective multicenter setting. Patients who reached normal expression of all seven genes by day 15 had excellent prognoses. Patients with a combination of cytological nonremission on day 28 and high-risk MRD (elevated expression of at least one marker on day 28) had an extremely poor prognosis (83). Furthermore, the timing of donor-lymphocyte infusions (DLIs) is crucial due to the absence of dependable indicators for posttransplant relapse status. Monitoring PRAME in the posttransplant phase can provide valuable insights into identifying the most suitable time for DLI administration.

## 7 Adoptive immunotherapy

The effectiveness of immunotherapeutic approaches, including monoclonal antibodies, cytokines, immunomodulatory agents, cellular immunotherapies such as vaccination, dendritic cell (DC) therapy, and T-cell activating antibodies such as immune checkpoint inhibitors, bispecific antibodies and chimeric antigen receptor T cells (CAR-T cells), is becoming increasingly evident (84). AML patients, however, do not have a widely expressed surface antigen that can be targeted by antibodies and CAR-T-cell treatments. The high expression of PRAME in leukemia cells and its silencing in normal hematopoietic cells have opened new possibilities for immunotherapy in AML.

### 7.1 Preclinical studies

#### 7.1.1 PRAME-specific T cells

The application of adaptive immunotherapy in AML is relatively limited to posttransplant donor-derived natural killer cells and CTLs (85), which identify antigens on leukemia cells. These antigens include minor histocompatibility antigens and LAAs (86). The PRAME protein is antigenic *in vitro* and stimulates the proliferation and activation of specific CD8^+^ CTLs in AML (87). PRAME-specific T cells can be generated either by stimulating T cells with PRAME and other TAAs or by transferring TCRs specific for PRAME to T cells (TCR-T), followed by ex vivo expansion and selection.

Expanding PRAME-specific T cells ex vivo requires the utilization of antigen-presenting cells (APCs), such as DCs or artificially engineered APCs (88, 89). AML-DCs expressing PRAME can elicit T-cell responses (90). The challenge of obtaining a large number of DCs for large-scale leukemia-specific T-cell generation has limited its clinical application. A method of ex vivo rapid expansion of T cells expressing the activation marker CD137 (4-1BB) was developed by Lee et al. after exposure to overlapping PRAME peptides (91). In addition, Koukoulias et al. proposed a unique “circular economy” model that generates billions of DCs through the repeated use of nontransplantable umbilical cord blood units, followed by the production of clinically relevant quantities of third-party, bivalent leukemia-specific T cells to target the PRAME (92). Moreover, exercise can enhance the *ex vivo* expansion of PRAME-specific CTLs from healthy adults without compromising cytotoxic function (93, 94). Currently, research has reported the *in vitro* and *in vivo* effects of amplified PRAME-specific CTLs, and we have summarized the relevant preclinical data in **Table 1**.

**Table 1:** Preclinical data of adoptive PRAME-specific T cells for AML.

Study	Treatment	In vitro efficacy	Animal model	In vivo efficacy
Matsushita 2001 (49)	PRAME-specific CTLs	Killing PRAME-positive leukemia cell line K562 and fresh leukemia cells from AML patient	N/A	N/A
Greiner 2006 (57)	PRAME-specific CTLs	Killing PRAME–peptide pulsed T2 cells and primary AML blasts with PRAME expression	N/A	N/A
van den Ancker 2013 (95)	PRAME-specific CTLs	Against AML cell lines K562 and patient-derived leukemia cells in a dose-dependent manner	N/A	N/A
Amir 2011 (96)	PRAME-specific allo-HLA restricted CTLs	Clearing primary AML cells	N/A	N/A
Quintarelli 2011 (97)	PRAME-specific CTLs	Against leukemic blasts and leukemic progenitor cells, do not affect normal hematopoietic progenitors	N/A	N/A
Schneider 2015 (79)	PRAME-specific CTLs	Against AML cell lines OCI-AML2, OCI-AML3, and patient-derived leukemia cells	Mice	Targeting AML stem cells
Hashimoto 2022 (98)	PRAME-specific T_H_1 cells	Induce cell-cycle arrest and senescence in AML cell lines Nomo-1, Kasumi, and fresh patient-derived AML blasts through combinative IFN-γ and TNF-α	N/A	N/A
Yao 2013 (99)	PRAME-specific CTLs	Killing AML cells THP-1 and enhanced by chidamide alone or combined with decitabine	N/A	N/A
Greiner 2022 (100)	PRAME-specific CTLs	Enhancing the effect of PRAME stimulated CTLs on LPC/LSC by nivolumab	N/A	N/A
Kirkey 2023 (47)	PRAME ^mTCR^CAR-T	Target-specific and HLA-mediated in vitro activity in OCI-AML2 and THP-1 cell lines, HLA-A2 cell lines expressing the PRAME antigen, and against primary AML patient samples	Mice	Potent leukemia clearance and improved survival

PRAME, preferentially expressed antigen in melanoma; CTLs, cytotoxic T lymphocytes; N/A, not available; AML, acute myeloid leukemia; IFN-γ, interferon gamma; TNF-α, tumor necrosis factor α; LPC/LSC, leukemic progenitor/stem cells; mTCR, TCR mimic; CAR-T, chimeric antigen receptor T cells.

PRAME-specific CTLs can lyse PRAME-positive leukemia cell lines and fresh leukemia cells (49, 57, 95, 96). In addition, PRAME-specific T cells also protect against LSCs/LPCs, which have been implicated in leukemia relapse (97). In a xenotransplant mouse model, PRAME-stimulated CTLs were shown to target AML stem cells, as reflected by delayed engraftment of leukemia cells (79). Interestingly, these PRAME-directed CTLs do not affect normal hematopoietic progenitors.

PRAME-specific T-cell responses can be enhanced by combination with other drugs. Hashimoto et al. reported that PRAME-specific T_H_1 cells induced senescence and cell cycle arrest in AML cell lines and fresh patient-derived AML blasts through the combination of IFN-γ and TNF-α (98). Yao et al. showed that PRAME-specific CTLs exhibited enhanced cytotoxicity to leukemia cells *in vitro* following treatment with either the HDAC inhibitor chidamide alone or in combination with the hypomethylating agent decitabine. These effects were achieved through mechanisms involving the upregulation of PRAME and CD86 in AML cells (99). Greiner et al. showed that T cells from AML patients could respond to LAAs (PRAME, WT1 and RHAMM) after stimulation. The presence of an anti-PD-1 blocking antibody improved T-cell-mediated antitumor responses against LPCs/LSCs (100). These studies provide a rationale for combining drugs with adoptive cell therapy to improve efficacy in clinical circumstances.

For PRAME specific TCR-T cells, Sailer et al. reported transducing a chimeric PD1-41BB receptor can enhance IFN-γ secretion, improve cytotoxic capacity, and prevent exhaustion *in vitro* without changing safety. Moreover, the addition of PD1-41BB could eradicate refrectory melanoma in mice that was resistant to TCR-T cells without PD1-41BB (101). The study supports the development of similar PRAME specific TCR-T cells for AML.

#### 7.1.2 Antibodies and CAR-T cells

**Figure 4:**
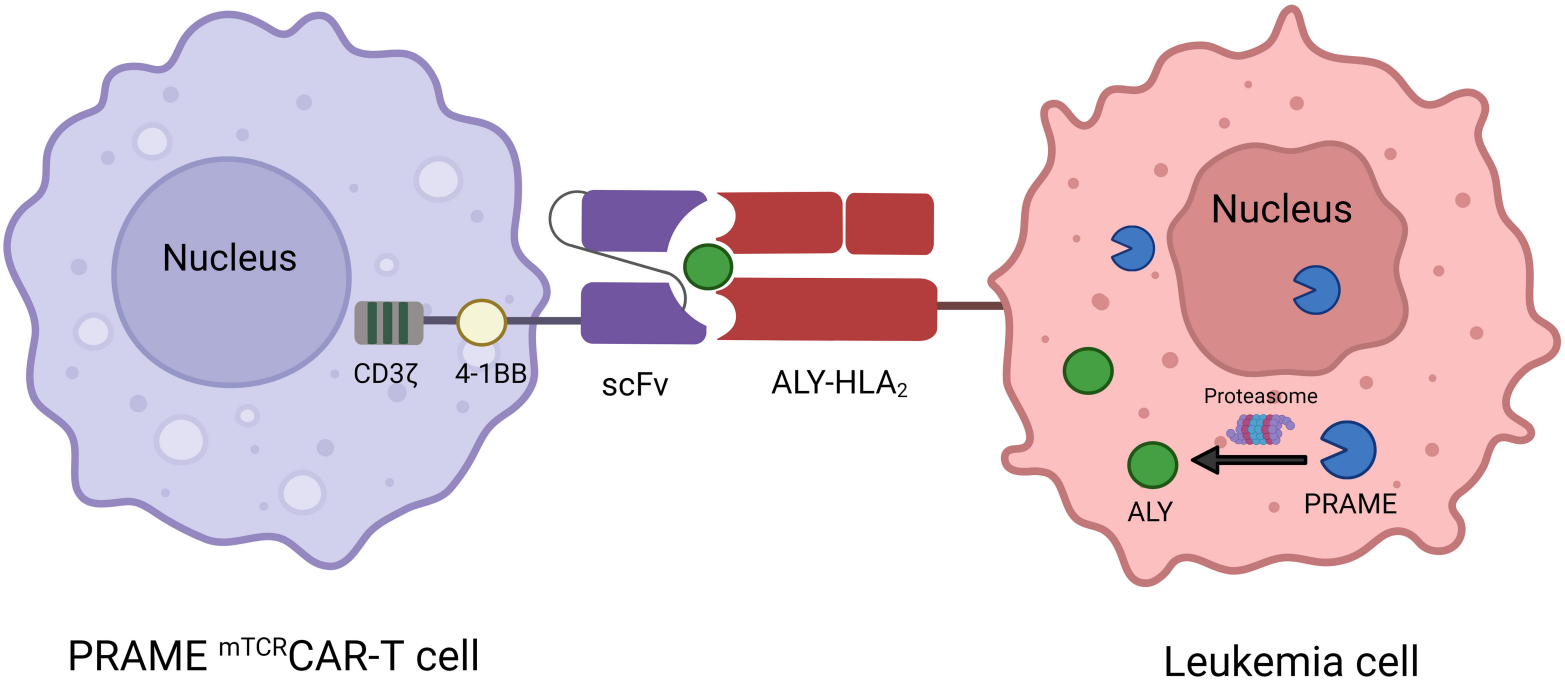
Mechanism of interaction between PRAME ^mTCR^CAR-T cells and PRAME+ leukemia cells. ScFv from Pr20 is used to construct PRAME mTCRCAR-T cells, which can specifically recognize the ALY-HLA-A2 complex after intracellular PRAME is processed into ALY by proteasomal enzymes.

The treatment of B-cell malignancies has significantly advanced with the success of CAR-T-cell therapy. Nevertheless, the application of CAR-T-cell therapy in AML is currently at an early stage and faces constraints due to the inherent diversity linked to AML and the absence of specific targets for therapeutic advancement. The present approaches employ lineage indicators such as CD33 and CD123 as targets for therapy, which, if successful, may result in myeloablation (102, 103). PRAME is highly expressed in AML and is absent in normal hematopoietic cells, which provides an effective target for CAR-T-cell therapy in AML. Unfortunately, PRAME is an internal protein, making it inaccessible to conventional CAR-T cells, which are limited to antigens on the cell surface. However, PRAME can be processed by the proteasome into four HLA-A*02:01–restricted epitopes, including the PRAME^100-108^ peptide VLDGLDVLL, the PRAME^142-151^ peptide SLYSFPEPEA, the PRAME^425-433^ peptide SLLQHLIGL and the PRAME^300–309^ peptide ALYVDSLFFL (ALY) (104). Rezvani et al. revealed that all four PRAME-derived peptides were immunogenic in HLA-A*0201-positive patients with AML, ALL and CML, but only ALY induced CD8+ T-cell responses (87). Chang et al. designed Pr20, an afucosylated Fc form of a TCR mimic (mTCR) human IgG1 antibody that can specifically recognize the cell surface ALY-HLA-A2 complex in PRAME+HLA-A2+ leukemia. Pr20 mediates antibody-dependent cellular cytotoxicity against PRAME+HLA-A2+ leukemias *in vitro* and is effective against AML xenograft models in mice, making Pr20 a potential therapeutic agent (13). Using the Pr20 monoclonal antibody sequence, Kirkey et al. created PRAME^mTCR^CAR-T cells to target the PRAME antigen in AML, and the scFv of CAR-T cells could specifically recognize and bind the ALY/HLA-A2 complex in PRAME+HLA-A2+ AML but not in PRAME-HLA-A2+ or PRAME+HLA-A2- AML (**Figure 4**). These cells showed specific activity against the target and were effective *in vitro* in OCI-AML2 and THP-1 cell lines, as well as in HLA-A2 cell lines expressing the PRAME antigen and primary AML patient samples. PRAME^mTCR^CAR-T cells effectively eliminated leukemia and enhanced survival in *in vivo* cell-derived xenograft models. Moreover, the cytolytic function of the PRAME^mTCR^CAR-T cells was enhanced through the application of interferon gamma to the targeted leukemia cells because interferon gamma can increase PRAME antigen expression (47). This study describes a novel adoptive cell therapy involving the use of PRAME with ^mTCR^CAR-T cells for the treatment of AML, which warrants further evaluation in clinical trials. In addition, further development of ^mTCR^CAR to target other intracellular AML-specific antigens in an HLA-restricted manner is needed.

### 7.2 Clinical trial

Relapse after intensified induction chemotherapy or allogeneic hematopoietic stem cell transplantation (allo-HSCT) is the main cause of death in AML patients. Unselected DLIs are used as rescue or maintenance therapies for AML patients due to the efficacy of graft-versus-leukemia (GVL). However, as DLIs are not leukemia specific, GVL frequently manifests as perilous graft-versus-host disease (GVHD) resulting from the simultaneous transmission of alloreactive lymphocytes. LAA-specific CTLs can selectively target leukemia cells, thereby exerting GVL effects without causing GVHD (88). PRAME specific T cells and PRAME vaccines are currently being extensively studied.

#### 7.2.1 PRAME-specific T cells

Several trials utilized PRAME specific T cells targeting multiple TAAs showed good safety and feasibility. To create a secure and efficient cellular treatment for individuals experiencing recurring AML/MDS, Lulla and colleagues developed a T-cell treatment that focuses on numerous LAAs, aiming to replicate the GVL effect caused by T cells from a donor while minimizing the chance of triggering GVHD. T cells with inherent TCRs targeting LAAs (PRAME, WT1, Survivin, and NY-ESO-1) were amplified from the PB of HSCT donors (NCT02494167). Products were successfully produced from 29 HSCT donors and have been proven to have multileukemia antigen specificity (mLST). Unlike DLIs, mLSTs specifically identify and eliminate leukemia antigen-expressing cells but exhibit no effect on normal cells derived from the recipient *in vitro*. Twenty-five participants in the trial who had AML/MDS after HSCT were given increasing amounts of these mLSTs (ranging from 0.5 to 10×10^7^ cells/m^2^). Infusions were well tolerated with no grade >2 acute or extensive chronic GVHD. The median LFS and OS were not reached after 1.9 years of follow-up (105). Kinoshita et al. assessed the safety and clinical results of 3 LAAs (PRAME, WT1, and Survivin) after administering a new T-cell treatment to patients with acute leukemia who experienced relapse or were at a high likelihood of relapse following allogeneic bone marrow transplantation (BMT) (NCT02203903). LAAs were targeted using lymphocytes obtained from BMT donors. LAA lymphocytes were administered to patients at doses ranging from 0.5 to 4×10^7^/m^2^. There were 23 recipients of BMT with relapsed/refractory (n = 11) and high-risk (n = 12) AML (n = 20) or acute lymphoblastic leukemia (n = 3). There were no instances of cytokine release syndrome or neurotoxicity observed among the patients, with only 1 patient experiencing grade 3 GVHD. Relapsed individuals demonstrated a 36% 1-year OS rate and a 27.3% 1-year LFS rate following LAA lymphocyte infusion. Patients with the worst prognosis who relapsed 6 months after the transplant had a postrelapse 1-year OS rate of 42.8% (n = 7). In 12 high-risk patients who received preemptive LAA infusion, the median survival was not reached. Furthermore, LAA lymphocytes remained detectable for a minimum of 12 months following infusion (106). Another trial with T cells targeting the same 3 LAAs (PRAME, WT1, and Survivin) enrolled 11 patients with AML or MDS after HSCT. The study has been terminated and no results were released (NCT04679194). In addition, Monzr et al. reported preliminary results of donor-derived CD8+ T cells targeting WT1, PRAME, and Cyclin A1 (NCT04284228) in relapsed AML after allogeneic HSCT. Five of 7 patients enrolled received three different dose levels: 50, 100 and 200 million. No GVHD, cytokine release syndrome (CRS) and neurotoxicity were observed while GVL effects were indicative, including decrease in blood transfusion, blast burden, myeloid sarcoma volume and increase in donor chimerism. Adoptive CD8+T cells proliferated rapidly and can be detected in blood and bone marrow (107).

However, clinical trials with PRAME specific TCR T cells are not going smoothly. Due to the sponsor’s discretion, 2 related phase I/II trials had terminated in the first stage. One trial involved 9 patients with relapsed or refractory AML(n=6), myelodysplastic syndrome and myeloproliferative neoplasm (MDS) (n=1) and multiple myeloma (n=2). The patients received one of three escalating dose levels (0.1,1,5) million PRAME TCR-transduced T cells/kg. One AML patient treated with 0.1 million achieved complete remission at week four, but disease progressed at week twelve. One patient with MDS/MPN treated with 5 million maintained stable during the 12-month study. Furthermore, TCR-T cells were detectable in peripheral blood of 6 of 8 patients in the first four weeks and persistent in the MDS/MPN patient at twelve months. Two patients experienced controllable CRS of Grade 1 and Grade 2, respectively. No neurotoxicity or dose-limiting toxicities were observed((NCT03503968) (108). Another trial involved 4 patients with relapsed AML and previously treated MDS. The patients received PRAME TCR-transduced T cells with 1.25 x 10^6^ cells/kg. Two patients reported one of following adverse events, including neutropenic fever, tachypnea, CRS, neurotoxicity, bacteremia, infection and orthostatic hypotension. No more information was posted in ClinicalTrials.gov (NCT02743611). CRS seems to be only in PRAME specific TCR T cells therapy rather than in PRAME specific T cells targeting multiple TAAs treatment. More studies with PRAME specific TCR T cells are needed to clarify its safety. The relevant clinical data of PRAME specific T cells was briefly summarized in **Table 2**.

**Table 2:** Clinical data of PRAME-specific T cells for AML.

Study	Trial status	Clinical phase	Patient number	Age (years)	Disease	Target	Treatment	Dose	Outcome	Adverse events
NCT02494167 (Lulla 2021) (105)	Recruiting	I	25	16–73	AML or MDS after HSCT	PRAME/WT1/Survivin/NY-ESO-1	Multi-TAA-specific T cells	0.5–10x10^7^ cells/m^2^	Not yet reached median LFS and OS at 1.9 years of follow-up	No grade >2 acute or extensive chronic GVHD
NCT02203903; (Kinoshita 2022) (106)	Recruiting	I	23(20 AML)	1–70	AL after HSCT	PRAME/WT1/Survivin	LAA-T	0.5–4x10^7^ cells/m^2^	One-year OS in patients who relapsed early post-BMT was 42.8% post-relapse, whereas 88.9% of evaluable high-risk patients were alive at 1 year	One patient developed grade 3 GVHD
NCT04284228	Active, not recruiting	I/II	5	N/A	Relapsed AML or MDS after HSCT	PRAME/WT1/Cyclin A1	Multi-TAA-specific CD8+ T cells	1–2×10^8^	improve GVL effect	No GVHD, CRS and neurotoxicity
NCT04679194	Terminated	I	11	N/A	AML or MDS after HSCT	PRAME/WT1/Survivin	Multi tumor-associated antigen T cells	N/A	N/A	N/A
NCT02743611	Terminated	I/II	4	39-72	Relapsed AML, previously treated MDS or metastatic uveal melanoma	PRAME	PRAME TCR gene modified T cells	1.25-5x10^6^ cells/kg	N/A	2 patients reported one of followings: neutropenic fever, tachypnea, CRS, neurotoxicity, bacteremia, infection, orthostatic hypotension
NCT03503968	Terminated	I/II	9(6 AML, 2 MM, 1 MDS/MPN)	N/A	Relapsed/refractory myeloid and Lymphoid Neoplasms	PRAME	PRAME TCR gene modified T cells	0.5-10 x10^6^ cells/kg	1 AML achieved CR at week 4 and progressed at week 12. 1 MDS/MPN remained stable	2 patients exhibited grade1-2 CRS, no neurotoxicity.

PRAME, preferentially expressed antigen in melanoma; AML, acute myeloid leukemia; MDS, myelodysplastic syndrome; HSCT, hematopoietic stem cell transplantation; TAA, tumor associated antigen; LFS, leukemia free survival; OS, overall survival; GVHD, graft versus host disease; LAA-T, T-cell therapeutic targeting 3 Leukemia associated antigens; GVL, graft versus leukemia; CRS, cytokine release syndrome; N/A, not available; MM, multiple myeloma; MDS/MPN, myelodysplastic syndrome and myeloproliferative neoplasm; CR, complete remission.

#### 7.2.2 PRAME vaccination

PRAME overexpression in AML provides a target for vaccine strategies. Steger et al. studied the expression of PRAME, WT1 and PR3 (proteinase 3) in AML. High expression was found in 87%, 81% and 55% of the patients with AML, respectively. Approximately 70% of the AMLs had overexpression of PRAME and WT1, and 45% of the AMLs had overexpression of all 3 TAAs. Furthermore, PRAME expression increased during the course of AML persistence. Consequently, it is reasonable to cover different peptides for AML immunotherapy with vaccines (109).

Currently, PRAME vaccination for AML are mainly DC-based. DCs effectively display antigens to CD8^+^ and CD4^+^ T lymphocytes via major histocompatibility complex (MHC) class I and II molecules, respectively. *Li et al.* isolated DCs from 5 AML patients and generated PRAME expressing DCs. The cells were infused four times at a biweekly interval and were well tolerated. A significant increase of granzyme B releasing CD8+ T cells specifically recognizing ALY was detected. In addition, elevation in Th1 cytokines and interferon gamma production was also observed (110). FDC101, a vaccine consisting of mature DCs (mDCs) loaded with autologous RNA and expressing two LAAs (PRAME and WT1), was administered to 20 AML patients in CR1 who were not eligible for allo-HSCT. Throughout the 24-month research duration, every administration included 2.5–5×10^6^ mDCs per antigen, given on a weekly basis until the fourth week, at the sixth week, and subsequently monthly. The treatment was well tolerated, with mild and temporary grade 1 reactions at the injection site. CR was achieved in 11 of the 20 patients (55%), and CR2 was achieved in 4 of the 6 relapsing patients (67%) who received allo-HSCT after salvage therapy. The OS rate at 5 years was 75% (111).Lichtenegger et al. performed a phase I study utilizing TLR7/8-mDCs that were transfected with RNA encoding PRAME and WT1 along with CMVpp65. AML patients with a high likelihood of recurrence who successfully achieved CR received 10 vaccinations within a span of 26 weeks. In 11/12 patients, a sufficient number and quality of DCs were generated, and 10 patients were vaccinated. The management was secure and led to regional inflammatory reactions. A significant increase in antigen-specific CD8^+^ T cells was observed for PRAME (4/10), WT1 (2/10), and CMVpp65 (9/10). The median RFS was 3 years, and the median OS was not achieved within a period of 3 years. In addition, immune responses can be enhanced through the combination of TLR7/8-mDCs and immunomodulatory agents such as hypomethylating agents or checkpoint inhibitors (112).

One trial involved synthetic peptides containing NY-ESO-1, MAGE-A3, PRAME and WT-1 for MDS and AML patients. Five MDS patients were actually enrolled. The peptides were injected subcutaneously after 6 cycles of azacitidine. No serious adverse events occurred. However, vaccine-specific immune response was also not detected. All patients progressed to AML after a mean time of 4.9 months from inclusion. Due to lacking immune response and potential benefit, the trial was terminated early (NCT02750995) (113). The relevant clinical data of PRAME vaccination was briefly summarized in **Table 3**. Overall, PRAME vaccination for AML demonstrates good safety profile, DCs-based strategies may play a role in triggering immunological responses. More prospective trials are needed to evaluate the efficacies for AML.

**Table 3:** Clinical data of PRAME vaccination for AML.

Study	Trial status	Clinical phase	Patient number	Age (years)	Disease	Target	Treatment	Dose	Outcome	Adverse events
Li. 2006 (110)	N/A	N/A	5	54-71	AML in a palliative setting	TAAs including PRAME	Autologous AML-DCs	4 injections with 5×10^6^, biweekly	Three patients remained stable condition for 5.5-13 months, 2 patients died from AML, increase in granzyme B releasing CD8+T cells and Th1 cytokines and CD4+ derived interferon gamma	Mild and transient injection site reactions
NCT02405338 (Fløisand. 2023) (111)	Completed	I/II	20	23–72	De novo AML in CR1	PRAME/WT1	RNA-loaded DC vaccine (FDC101)	2.5–5×10^6^ DCs/antigen	Maintain CR: 55%; achieved CR2: (4/6); 5-year OS: 75% (95% CI: 50–89)	Mild and transient injection site reactions
NCT01734304 (Lichtenegger 2020) (112)	Completed	I	13	44–79	AML in CR	PRAME/WT1/CMVpp65	TLR 7/8-matured RNA-transduced DCs	10 vaccinations over 26 weeks	Median OS: 1057 days; RFS: 1084 days	One grade 3: pyrexia
NCT02750995 (Staffan 2020) (113)	Completed	I	5 (MDS)		MDS, AML	NY-ESO-1, MAGE-A3 and PRAME. WT-1	Azacitidine followed by peptides injection	N/A	No vaccine-specific immune response	One grade 4: neutrophil count decrease; 2 grade 3: platelet and neutrophil count decrease

PRAME, preferentially expressed antigen in melanoma; AML, acute myeloid leukemia; N/A, not applicable; TAA, tumor associated antigen; DCs, dendritic cells; CR, complete remission; TLR, Toll-like receptor; OS, overall survival; MDS, myelodysplastic syndrome.

## 8 Conclusion

PRAME is highly expressed in leukemia cells and LPCs/LSCs but not in HSPCs, suggesting that it is a preferred target for AML treatment. PRAME is a useful molecular marker for detecting remission and monitoring MRD in AML patients. During the posttransplant period, monitoring PRAME levels will indicate the optimal timing for DLIs. Although the cellular mechanism of action of PRAME requires further investigation, a deeper understanding of its function is expected to provide a new perspective on adaptive T-cell immunotherapy for AML. An increasing number of studies on PRAME-specific immunotherapy, especially in combination with demethylating agents, HDAC inhibitors or allo-HSCT, are expected to emerge.

## Author contributions

JJY: Data curation, Formal analysis, Resources, Software, Visualization, Writing – original draft. MRC: Visualization, Writing – review & editing. JY: Visualization, Writing – review & editing. HBM: Conceptualization, Data curation, Formal analysis, Funding acquisition, Resources, Software, Visualization, Writing – original draft, Writing – review & editing.
